# Research on B_4_C/PEEK Composite Material Radiation Shielding

**DOI:** 10.3390/polym16202902

**Published:** 2024-10-15

**Authors:** Hongxia Li, Hongping Guo, Hui Tu, Xiao Chen, Xianghua Zeng

**Affiliations:** 1 College of Electrical, Energy and Power Engineering, Yangzhou University, Yangzhou 225009, Chinamz120221223@stu.yzu.edu.cn (H.T.); xiaoc0222@163.com (X.C.); 2 Innovation Center for Radiation Application, Beijing 102413, China

**Keywords:** radiation shielding, simulation, hardening component, B_4_C/PEEK, composite material

## Abstract

There are various types of charged particles in the space environment, which can cause different types of radiation damage to materials and devices, leading to on-orbit failures and even accidents for spacecraft. Developing lightweight and efficient radiation-shielding materials is an effective approach to improving the inherent protection of spacecraft. The protective performance of different materials against proton and electron spectra in the Earth’s radiation belts is evaluated using a Geant4 simulation. Based on the simulation results, suitable hardening components were selected to design composite materials, and B_4_C/PEEK composites with different B_4_C contents were successfully prepared. The experimental results demonstrate that the simulated and experimental results for the electron, proton and neutron shielding performance of the B_4_C/PEEK composites are consistent. These composites exhibit excellent radiation shielding capabilities against electrons, protons and neutrons, and the radiation protection performance improves with increasing B_4_C content in the B_4_C/PEEK composite materials.

## 1. Introduction

In the space environment, there exist various types and energies of charged particles, mainly electrons and protons, which can cause radiation damage to spacecraft. As human space activities expand, spacecraft face increasingly stringent tests and challenges in terms of on-orbit service life and reliability [1]. Developing lightweight and efficient radiation-shielding materials is an effective approach to provide intrinsic physical protection against radiation damage to spacecraft materials and components [2]. The radiation damage to spacecraft materials and components can be effectively reduced by directly blocking and absorbing the energy of external radiation particles by installing a certain thickness protective layer on the surface and around the spacecraft materials and components that are vulnerable to radiation damage [3]. The development of lightweight and efficient radiation protection composite materials is one of the effective ways to reduce the radiation damage of airborne electronic components.

Among the top ten advances in the field of advanced materials in the foreign defense science and technology annual report for 2020, the research conducted at North Carolina State University in the United States reported on a polymer-based radiation protection composite material embedded with bismuth trioxide (Bi_2_O_3_) particles [4]. This radiation protection material is mainly based on the ultraviolet irradiation curing method, composed of 44% bismuth trioxide added with polymethyl methacrylate (PMMA), which can effectively shield ionizing radiation such as gamma rays. It has the advantages of high strength, light weight, non-toxic and low cost, and it is a potential substitute for traditional radiation protection materials such as lead and aluminum. It can be used in radiation protection for human space exploration, radiation therapy, medical imaging, and other applications [5,6]. This indicates that polymer-based radiation protection composite materials are receiving high attention from foreign researchers and are one of the current hotspots in radiation protection material research.

Domestic researchers, such as Rui Erming et al. [7], incorporated carbon nanotube reinforcement elements into Low-Density Polyethylene (LDPE) to produce radiation-protection composite materials. Their study revealed that under the same mass thickness, MWCNTs/LDPE composite materials exhibited superior proton and electron radiation shielding capabilities compared to metallic aluminum. The high hydrogen content of LDPE polymers contributes to enhanced radiation protection performance. However, LDPE itself possesses weak radiation resistance, leading to susceptibility to damage in LDPE-based composite materials after radiation exposure.

Studies have shown that boron is a good neutron-moderating element. Many scholars have added B_4_C with chemical properties to aluminum, rubber and other polymer materials to prepare composite materials to improve neutron protection properties. Jaewoo Kim et al. [8] prepared nano-scale boron carbide (B_4_C) and boron nitride (BN) powders using the ball milling method, and mixed the obtained micron powder and nano-powder with high-density polyethylene (HDPE), respectively, using a melt blending process, and then prepared sheet composite materials by hot pressing.

In recent years, it has become a research hotspot in the field of aeronautics and astronautics to process new polymeric radiation-shielding materials by adding nanoparticles with excellent radiation-shielding properties into specific polymer substrates. Poly ether ether ketone (PEEK) boasts exceptional high-temperature resistance, aging resistance, mechanical properties, insulation stability, and radiation resistance, making it widely applicable in aerospace [9,10,11,12].

With the development of nanotechnology, micro–nano particle units and polymer matrix composites with good radiation resistance provide a new way to develop lightweight and efficient space radiation protection materials [13,14,15,16]. This includes incorporating high atomic number elements (excluding lead), metal oxides, and graphite nanofibers (or nanotubes) as secondary elements into polymer matrices [17]. Compared to metallic lead, polymer-based composite materials prepared with these elements demonstrate significant lightweight and efficient characteristics [18,19]. Additionally, exploratory work on adding other elements is underway but it has yet to meet the application requirements for space radiation protection [20]. The key technological challenge in the development of polymer-based radiation protection materials lies in uniformly incorporating micro–nano level particle materials into radiation-resistant polymer matrices and producing lightweight structures with specific forms [21]. Research on preparing radiation-resistant composite materials based on PEEK is still in its early stages. There is a lack of necessary design guidelines for radiation protection composite materials, and related manufacturing process technologies need to be explored from scratch [22]. In order to provide theoretical guidance and technical support for the development of new polymer radiation protection composites, the design concept, preparation and characterization of PEEK-based radiation protection composites reinforced by nanoparticles were deeply studied [23,24,25].

In essence, this means that basic principles and guidelines for the design of radiation-resistant composites based on PEEK need to be established. Researchers would need to explore various methods to incorporate nanoparticles into PEEK matrices effectively. This involves investigating the properties and behavior of these materials under radiation exposure, as well as developing appropriate manufacturing processes to ensure the uniform distribution and effective reinforcement of nanoparticles within the polymer matrix. Additionally, comprehensive characterization techniques are necessary to evaluate the performance of these composites in terms of radiation-shielding efficacy, mechanical properties, durability, and other relevant factors. Overall, this research direction aims to lay the groundwork for the development of advanced radiation protection materials using polymer composites based on PEEK.

## 2. Radiation Protection Composite Material Simulation, Calculation, and Design

### 2.1. Calculation of Shielding Effectiveness for Different Types of Materials and Selection of Matrices

**Figure 1:**
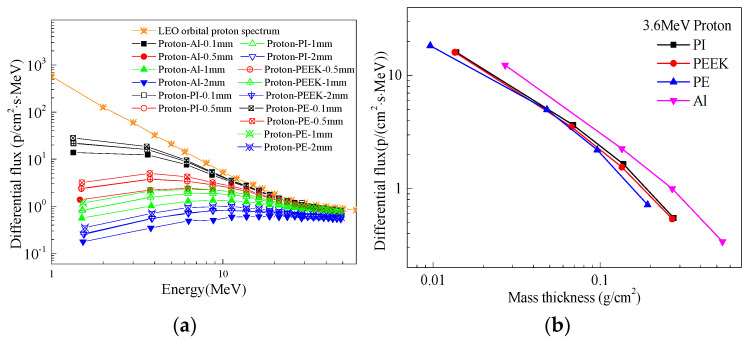
Differential cumulative flux spectra of LEO orbital protons protected by different materials and the same mass thickness: (**a**) differential cumulative flux spectra after different material and thickness protection; (**b**) proton protection effect of different materials with the same mass thickness.

Figure 1 illustrates the proton-shielding effectiveness of different thicknesses of traditional space-shielding material Aluminum (Al) and promising polymer materials Polyimide (PI), Poly ether ether ketone (PEEK), and Polyethylene (PE) for Low Earth Orbit (LEO) radiation belts (orbit altitude 200–600 km, inclination approximately 45°). Figure 1b represents the differential cumulative fluence for different materials at the same mass thickness. As observed from the graph, a significant reduction in the differential cumulative fluence compared to the LEO orbit spectrum, with better shielding effects achieved with thicker shielding layers. When comparing materials at the same thickness and proton energy, the proton differential cumulative fluence after polymer material shielding is slightly higher than that after Aluminum shielding, indicating that Aluminum provides better proton-shielding effects compared to polymers such as PE, PEEK, and PI at the same thickness. However, Aluminum, being a metallic material, is heavier and less conducive to spacecraft weight reduction. As shown in Figure 1b, calculations reveal that at the same mass thickness, the proton fluence after Aluminum shielding is higher than that after polymer material shielding, suggesting that polymer materials provide better proton-shielding effects than Aluminum at the same mass thickness. Figure 1a indicates that PE exhibits slightly better shielding effectiveness than PI and PEEK, but aromatic PEEK and PI demonstrate significantly superior comprehensive properties in terms of mechanics, thermal, and electrical characteristics, particularly in their ability to withstand radiation damage, which surpasses that of PE. Moreover, at higher proton energies, the shielding effectiveness of Aluminum and various polymer materials is comparable.

**Figure 2:**
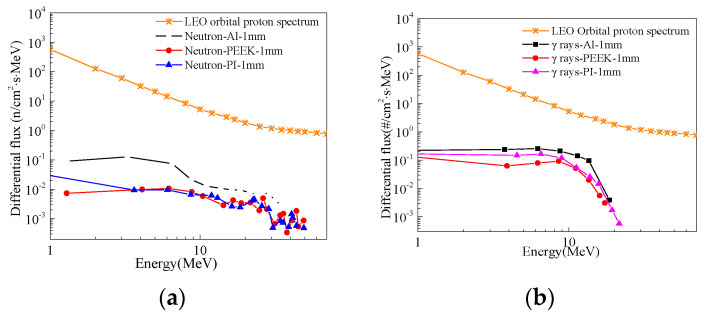
Differential cumulative flux spectra of secondary neutrons and gamma rays after LEO orbital protons pass through protective layers of different thicknesses: (**a**) secondary neutrons; (**b**) *γ* rays.

Figure 2 shows the differential cumulative fluence spectra of secondary neutrons and γ-rays after proton incidence through various thicknesses of shielding layers in the Low Earth Orbit (LEO). The depiction in the figure reveals that proton incidence on different shielding materials leads to the generation of secondary particles such as neutrons and γ-rays through nuclear spallation. The differential cumulative fluence of neutrons and secondary γ-rays produced by polymer materials is significantly lower compared to metallic Aluminum (Al), highlighting the effective reduction of secondary particle production by protons through the use of polymer materials. Research suggests that PEEK sheets exhibit strong resistance to radiation damage and show promise as a matrix material for polymer-based radiation protection. Considering these findings, PEEK is selected as the matrix for polymer-based radiation protection materials. However, it is acknowledged that a single material may not fully meet the varied spectrum of radiation protection requirements in space, underscoring the need for the development of lightweight and efficient radiation protection composite materials. Moreover, studies have indicated that composite materials created by integrating nanoparticles into polymer matrices contain numerous interfaces that contribute to improving the radiation protection performance of the composites.

### 2.2. Calculation of Protective Effect of Composite Materials and Selection of Reinforcement Components

**Figure 3:**
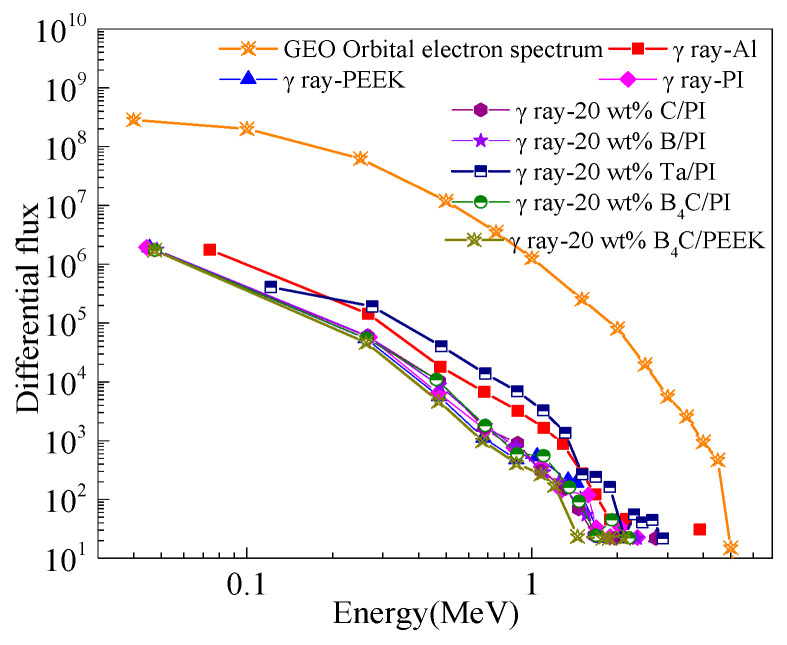
The differential cumulative flux spectra of the second gamma of GEO orbital electrons through 2 mm protective layer of different materials.

As shown in Figure 3, the differential cumulative flux spectra of secondary gamma photons after different 2 mm material shielding layers in the GEO orbit are depicted. It can be observed from the graph that the secondary particles produced by the 20 wt% Ta/PI composite material are more than those produced by the metal Al. Therefore, metallic Ta is excluded as a filler. The B_4_C/PEEK composite has a lower secondary photon differential cumulative flux. Based on the above factors, B_4_C was selected as the strengthening component to prepare a new radiation-shielding composite with different addition amounts of B_4_C/PEEK.

### 2.3. B_4_C/PEEK Protection Effect Calculation

**Figure 4:**
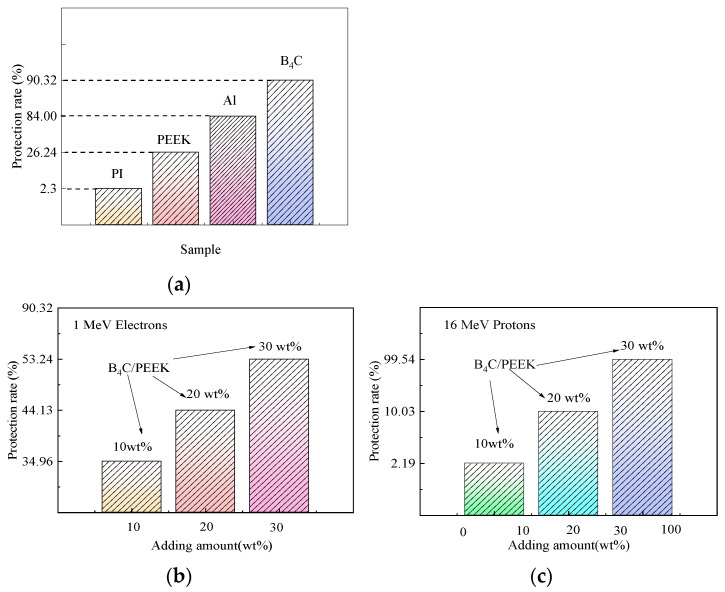
Simulation calculation results of 1 MeV electron and 16 MeV proton protection rate of materials: (**a**) protective effect of different materials; (**b**) electronic protective effect of B_4_C/PEEK at different B_4_C addition levels; and (**c**) 16 MeV proton protective effect of B_4_C/PEEK at different B_4_C addition levels.

Figure 4 illustrates the simulated results of the protective effects against 1 MeV electrons for different materials. In the graph, the thicknesses of PEEK, Al, and B_4_C materials are 2 mm, while the PI material has a thickness of 25 μm. Therefore, PI exhibits lower electron radiation protection compared to PEEK. It can be observed from the graph that B_4_C offers the best electron protection, followed by metallic aluminum, then polymer PEEK and PI materials. However, both B_4_C and metallic aluminum generate secondary particles after being irradiated by charged particles, and their high densities are unfavorable for spacecraft weight reduction.

By adding varying amounts of B_4_C to the radiation-resistant PEEK matrix, lightweight and efficient radiation-shielding B_4_C/PEEK composite materials can be obtained. Adding too much B_4_C adversely affects the mechanical properties of the composite material, while adding too little results in inadequate protection. Therefore, B_4_C filler addition amounts of 10 wt%, 20 wt%, and 30 wt% are designed for B_4_C/PEEK composite materials.

Simulations of radiation protection rates against 1 MeV electrons and 16 MeV protons for different B_4_C addition levels in B_4_C/PEEK composite materials are shown in Figure 4b,c. The protection rate is calculated in the simulation by the number of incoming particles minus the number of detected particles divided by the number of incoming particles, multiplied by 100%. The results showed that the protection rate of B_4_C against 1 MeV of electron radiation was significantly improved after adding B_4_C to B_4_C/PEEK composites, and the greater the amount of B_4_C added, the better the protection effect. This can be compared with experiments on electronic radiation protection. When the incident proton energy is high, such as 100 MeV, B_4_C/PEEK composite materials with different B_4_C addition amounts cannot completely shield them. However, when the incident proton energy is lower, such as 3 MeV and 10 MeV, B_4_C/PEEK composite materials can completely shield them. After adding B_4_C, the proton radiation protection rate of B_4_C/PEEK composite materials significantly increases with increasing B_4_C addition after irradiation with 16 MeV protons.

## 3. Preparation and Characterization of Experimental Materials

### 3.1. Test Materials and Equipment

The polyether ether ketone (PEEK) material used in the experiment, with a density of 1.35 g/cm^3^ and a thickness of 50 μm, is sourced from Victrex plc, Central Lancashire, UK. The boron carbide (B_4_C) powder, comprising 95%, is sourced from the School of Chemical Engineering at Harbin Institute of Technology. The precision electronic balance, model number 124-1CN, is sourced from Suzhou SANS Instrument Co., Ltd., Suzhou, China. The electrically heated constant temperature drying oven, model number 500-02, is sourced from Hu Yue Instrument Equipment Factory in Shaoxing, Zhejiang Province, China. The planetary ball mill, model number YXQM, is sourced from Changsha Miqi Instrument Equipment Co., Ltd., Changsha, China. The high-temperature press machine, model number HBSCR-100T, is sourced from Qingdao Bohua Technology Co., Ltd., Qingdao, China. 

### 3.2. B_4_C/PEEK Preparation Technology

**Figure 5:**
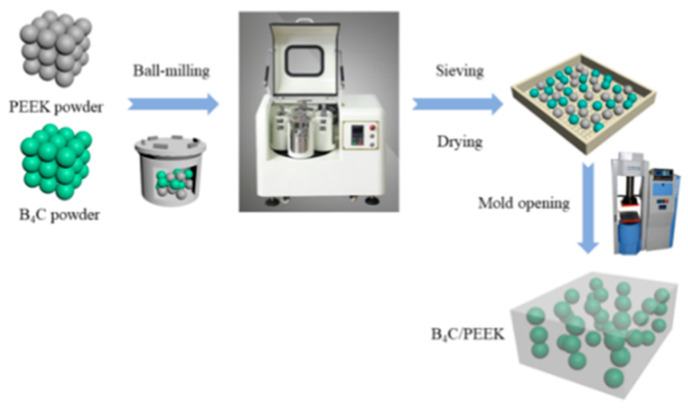
Preparation flow diagram of B_4_C/PEEK composite materials.

Using compression molding, B_4_C/PEEK composite materials with different B_4_C addition levels were prepared. To achieve excellent radiation-shielding performance, the key lies in enhancing the compatibility between the B_4_C component and the PEEK matrix, as well as improving the dispersion of the added B_4_C particles in the matrix. A certain amount of B_4_C reinforcing component and a specific quantity of 200-mesh PEEK matrix particles were weighed using an electronic balance. After thorough mixing, the mixture was placed into a planetary ball mill for grinding. Following grinding, the B_4_C and PEEK mixture exhibited exceptional homogeneity, with both the B_4_C component and the PEEK matrix ground into nanoscale sizes. The preparation process for B_4_C/PEEK using compression molding is illustrated in Figure 5. By separately weighing different amounts of B_4_C reinforcing component and employing the aforementioned preparation technique, B_4_C/PEEK composite materials with B_4_C addition levels of 10 wt%, 20 wt%, and 30 wt% were successfully prepared.

### 3.3. Characterization Method

The testing of electron radiation protection performance was conducted at the Institute of Technical Physics, Heilongjiang Academy of Sciences, using a high-frequency high-voltage electron accelerator. The energy of the electron beam was 1 MeV. The proton radiation protection testing was carried out on the R20 branch of the HI-13 serial accelerator at Beijing, where the beam spot area was 50 mm × 50 mm, and the non-uniformity of the beam spot distribution was better than 10. Various energy single-energy proton beams (energy resolution less than 10^−3^) were used to irradiate B_4_C/PEEK test specimens, with energy ranging from 12 MeV to 20 MeV. During irradiation, the proton beam intensity was controlled to be around 2.0 × 10^4^ p/s, and the irradiation time for each individual specimen exceeded 100 s. The neutron radiation protection testing was conducted at the Small Angle Spectrometer of the Dongguan Chinese Spallation Neutron Source, using B_4_C/PEEK composite material samples.

## 4. Radiation Protection Result

### 4.1. Electronic Radiation Protection Effect

The electronic radiation protection properties of the B_4_C/PEEK composites were tested at a 1 MeV electron irradiation dose of 2 × 10^14^ e/cm^2^. According to the absorbance data obtained by the thin film dosimeter and compared with the standard provided by the measuring institution, the absorbed dose of irradiation is calculated using Equation (1).

(1) $$D=5.45023X^{1.15071}\;X=\frac{A-A_{0}}{42.5}\times 1000$$

*D*—Radiation absorbed dose of dose tablets placed at different thicknesses,

*X*—Absorbance per unit thickness of the dose tablet,

*A*—The absorbance value of a radiation-changing film dose-piece after irradiation,

*A*_0_—The background absorbance value of the radiation-changing film dose tablet before irradiation.

**Figure 6:**
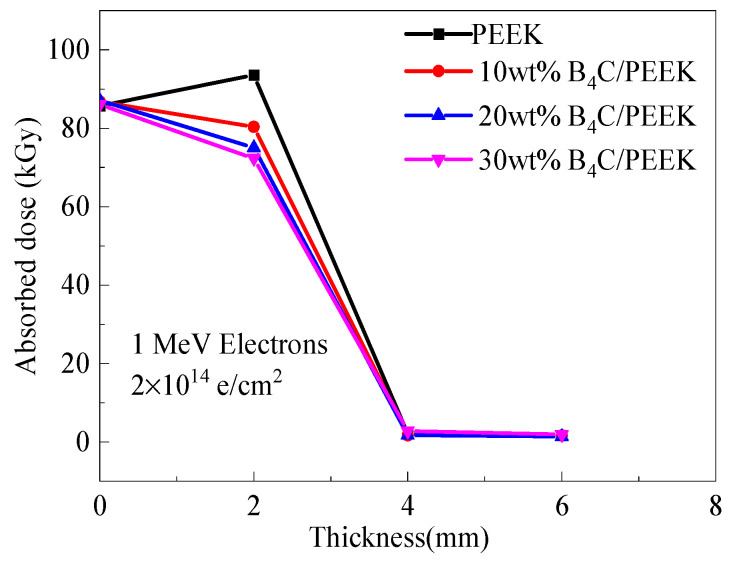
Distribution curves of film dose tablets along the thickness of B_4_C/PEEK under electron irradiation.

The smaller the absorbed dose measured in the experiment, the better the electron radiation protection effect of the composite material being tested. Figure 6 shows the absorbed dose distribution curves of the B_4_C/PEEK composites with different B_4_C addition amounts along the depth of the film dosimeter under electron irradiation. It can be observed that the absorbed dose of the PEEK material’s thin film dosimeter is higher than that of the surface dosimeter at a depth of 2 mm. This difference occurs because 1 MeV electrons penetrate the dosimeter and the material at a depth of 0mm, while the electrons in PEEK materials have a range of about 2 mm, allowing the deposited energy to accumulate in the PEEK material. Therefore, with changes in electron energy upon entering the material and the generation of secondary particles, an increase in dose occurs at the depth of 2 mm.

The absorbed doses of B_4_C/PEEK composite materials with different B_4_C addition levels are lower than those of surface dosimeters and significantly lower than the doses absorbed by dosimeters at 2 mm depth in PEEK. Additionally, they are lower than the absorbed doses of B_4_C/PEEK surface dosimeters, indicating the protective effect of B_4_C/PEEK composite materials. This suggests that with an increase in the B_4_C addition level, the electron radiation protection effect strengthens.

When the total shielding layer thickness is 4 mm, the dosimeter test results indicate an absorbed dose of approximately 1.5 kGy for electron irradiation. This is the background absorbed dose for the dosimeter before irradiation, indicating that the electrons are completely shielded, and the transmittance is 0. Therefore, the thickness of the material significantly affects the electron radiation protection effect.

### 4.2. Proton Radiation Protection Effect

**Figure 7:**
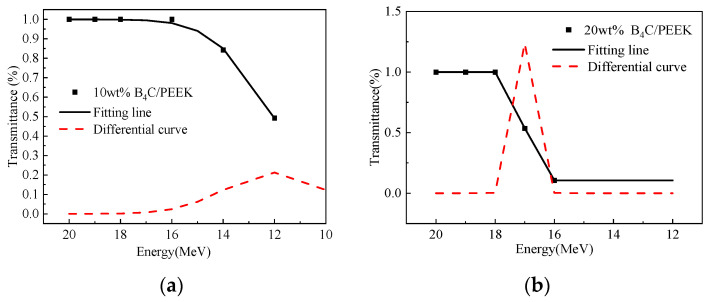
Proton transmittance of B_4_C/PEEK composites changes with energy: (**a**) 10 wt% B_4_C/PEEK, (**b**) 20 wt% B_4_C/PEEK.

The proton transmission rate curves with energy variation for composite materials with different B4C addition levels are shown in Figure 7. In Figure 7a, the sample corresponds to 10 wt% B4C/PEEK. It can be observed from the graph that when the energy exceeds 14 MeV, the incoming protons mostly penetrate through, and the proton transmission rate significantly decreases as the energy decreases, indicating a noticeable increase in protection. At 12 MeV, the proton transmission rate decreases to 52%. Based on this trend, it is inferred that for complete shielding of incoming protons, their energy should be below 10 MeV. The curve obtained by fitting the data points follows the trend of decreasing protection with increasing energy, indicating the correctness of the experimental results. The red dashed line in the graph represents the differential curve of the fitted curve, indicating the cutoff energy range of the material. Figure 7b illustrates the proton transmission rate curve with energy variation for 20 wt% B4C/PEEK composite material. It shows a significant decrease in proton transmission rate with decreasing energy, with protons below 16 MeV being essentially shielded and those above 18 MeV being mostly penetrable. This indicates an average cutoff energy of 17 MeV for this composite material. Comparing the results of proton radiation protection for composite materials with 10 wt% and 20 wt% B4C additions reveals a significant increase in the energy of shielded protons, from 10 MeV to 16 MeV. Therefore, B4C/PEEK composite materials with added B4C components exhibit significantly enhanced proton-shielding effectiveness.

### 4.3. Neutron Radiation Protection Effect

**Figure 8:**
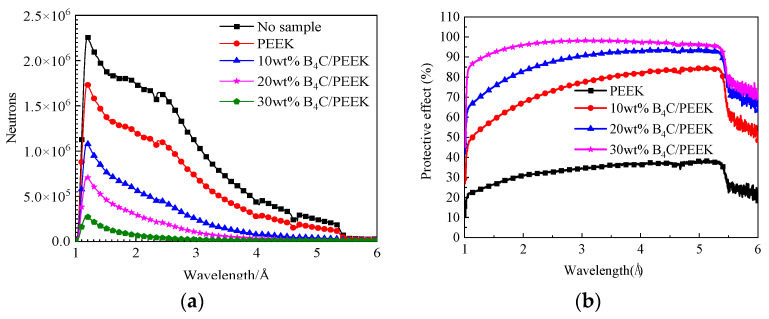
Neutron protection curves of B_4_C/PEEK composite materials with different B_4_C addition amounts: (**a**) variation curves of neutron count and wavelength; (**b**) change curves of wavelength and protection effect.

The neutron radiation protection testing curves for PEEK and B_4_C/PEEK composite materials with different B_4_C addition levels are shown in Figure 8. In Figure 8a, the relationship between neutron counts and wavelength is shown, while Figure 8b illustrates the relationship between protection effectiveness and wavelength. Formula (2) calculates the relationship between neutron flight time and neutron wavelength:

(2) $$\tau =\frac{m_{n}\lambda L}{h}$$

*h*—Planck’s constant,

*m_n_*—Mass of neutron,

*τ*—Flight time,

*L*—The distance the neutron travels from the chopper to the GEM detector.

According to Equation (2), the relationship between flight time and neutron counts can be transformed into the relationship between neutron wavelength and neutron counts, as shown in Figure 8. In Figure 8a, the neutron counts versus wavelength relationship for B_4_C/PEEK composite materials with different B_4_C addition levels are illustrated, while Figure 8b displays the relationship between protection effectiveness and wavelength. From the graphs, it can be observed that PEEK material exhibits some level of neutron protection effectiveness. After incorporating B_4_C reinforcing components, the neutron protection effectiveness of the composite material significantly increases, with greater addition amounts resulting in better neutron shielding. When the B_4_C addition level reaches 30 wt%, the neutron counts of the sample approach zero for neutron wavelengths greater than 3.1 Å.

Comparing the neutron counts of composite materials with different B_4_C contents reveals that the critical wavelength at which neutrons are completely shielded decreases with the B_4_C addition level increase. Additionally, it can be observed that the neutron count peaks around 1 Å wavelength and around 2.5 Å wavelength decrease with increasing B_4_C content in the sample, and the critical value at which neutrons are completely shielded shifts towards smaller angles with increasing B_4_C addition, indicating a significant enhancement in neutron protection effectiveness after adding B_4_C components. This is mainly attributed to the high slow neutron absorption cross-section of the ^10^B element in B_4_C, making it easier for larger-wavelength neutrons to be absorbed by the B_4_C/PEEK composite material. For B_4_C/PEEK composite materials, when the B_4_C addition level increases from 10 wt% to 30 wt%, a significant number of neutrons with wavelengths of 2.5 Å and longer are absorbed. This results in a relatively lower count rate of the GEM detector per unit time, leading to a decrease in count peaks.

## 5. Conclusions

The Geant4 program simulation results indicate that PEEK has better radiation protection performance against charged particles than aluminum at the same mass thickness. The addition of B_4_C components effectively enhances the radiation protection performance of polymer-based composite materials. By adjusting the process parameters, B_4_C/PEEK radiation protection composite materials with different B_4_C addition levels were successfully prepared.

The simulation results of electron and proton radiation protection of B_4_C/PEEK composites are consistent with the experimental results, showing excellent proton radiation protection ability. In addition, with the increase in B_4_C addition, the radiation protection performance improved. The electron, proton and neutron radiation protection properties of B_4_C/PEEK composites were enhanced with the increase in B_4_C content. When the doping amount of B_4_C is 30 wt%, the electron and neutron protection performance of the composite is the best.

## Data Availability

The original contributions presented in the study are included in the article, further inquiries can be directed to the corresponding author.
